# Influence of preoperative serum creatinine level and intraoperative volume of contrast medium on the risk of acute kidney injury after transfemoral transcatheter aortic valve implantation: a retrospective observational study

**DOI:** 10.1186/s13104-019-4527-2

**Published:** 2019-08-05

**Authors:** Daisuke Miura, Yasutaka Yamada, Shinichiro Kusaba, Eijiro Nogami, Junji Yunoki, Yoshiko Sakamoto, Yutaka Hikichi, Koichi Node, Yoshiro Sakaguchi

**Affiliations:** 1 Department of Anesthesiology, Saga University Medical Hospital, Saga, Japan; 2 Department of Thoracic and Cardiovascular Surgery, Saga University Medical Hospital, Saga, Japan; 3 Department of Cardiology, Saga University Medical Hospital, Saga, Japan

**Keywords:** Transfemoral transcatheter aortic valve implantation, Acute kidney injury, Serum creatinine, Contrast medium

## Abstract

**Objective:**

This study aimed to determine if contrast medium volume (CMV) is a risk factor for acute kidney injury (AKI) during transcatheter aortic valve implantation (TAVI) via a transfemoral approach performed without major complications. All TAVI procedures performed at our institution between March 2014 and March 2018 were retrospectively reviewed. AKI was diagnosed using the Acute Kidney Injury Network classification based on the Valve Academic Research Consortium-2 definition. Procedures performed via a transapical approach and those in which circulatory dynamics failed intraoperatively were excluded.

**Results:**

Eighty-one (96.4%) of 100 patients scheduled for TAVI were enrolled; seven (8.6%) developed AKI and 74 (91.4%) did not. The serum creatinine (SCr) level was significantly higher (p < 0.05) and the estimated glomerular filtration rate was significantly lower in the AKI group (p < 0.05). The CMV was significantly higher in the AKI group (103 ml vs 84 ml, p < 0.05), as was the CMV × SCr/BW value (3.34 vs 1.49, p < 0.01). The area under the curve for CMV × SCr/BW was 0.9228 and the cut-off value was 2.99. The CMV, SCr, and estimated glomerular filtration rate affect the likelihood of AKI after transfemoral TAVI and a CMV × SCr/BW value > 2.99 accurately predicts AKI.

**Electronic supplementary material:**

The online version of this article (10.1186/s13104-019-4527-2) contains supplementary material, which is available to authorized users.

## Introduction

Acute kidney injury (AKI) occurs in up to 30% of cases after cardiac surgery [1]. AKI after conventional cardiac surgery or percutaneous coronary intervention is associated with prolonged hospitalisation and increased mortality [2–6]. AKI can be caused by nephrotoxins, hypoxia, mechanical trauma, inflammation, cardiopulmonary bypass, and hemodynamic instability, and the risk of its occurrence may be affected by the choice of fluids, vasoactive agents, and transfusion strategy [7]. Contrast-induced nephropathy (CIN) is a type of AKI that occurs after administration of contrast medium and is usually reversible. The pathogenesis of CIN is uncertain but is thought to involve hypoxic injury and generation of free radicals [8]. The contrast medium volume (CMV) is considered to be a major risk factor for AKI [9–14]. Several studies have demonstrated an association between a CMV × serum creatinine (SCr)/body weight (BW) value > 5.0 and an increased risk of AKI or need for dialysis after percutaneous coronary intervention [11–14].

Transcatheter aortic valve implantation (TAVI) is preferred in patients with severe aortic stenosis who are not surgical candidates; however, AKI reportedly occurs in 8.3–58% of cases [15–21]. Predictors of AKI after TAVI include the baseline creatinine concentration [19, 22, 23], blood transfusion [15, 24], a transapical (TA) approach [21, 25], peripheral vascular disease [26], the EuroSCORE (Logistic European System for Cardiac Operative Risk Evaluation) [24], diabetes mellitus [23, 26] and use of a contrast agent [27]. However, the impact of CMV on the risk of AKI after TAVI remains controversial; although most studies have not shown a significant effect [15, 16, 19, 21, 28, 29], there are data suggesting that a higher dose might have a negative impact [30]. There are several explanations for these conflicting findings. First, the definition of AKI in studies of AKI after TAVI lacks standardisation [31] and second, most studies have included the TA approach, which is a known risk factor for AKI after TAVI [15, 17, 21, 25]. In this analysis of the influence of renal function-based contrast dosing on the risk of AKI, defined according to the VARC-2 criteria [32], we only included patients in whom transfemoral (TF) TAVI had been performed and who did not have major perioperative complications.

## Main text

### Study population

Patients who underwent transfemoral TAVI for severe aortic valve stenosis at our institution between March 2014 and March 2018 were considered for enrolment. Patients who underwent TA or trans-subclavian TAVI were excluded, as were those who developed major perioperative complications. The study protocol was approved by our institutional review board. The requirement for informed consent was waived.

### TAVI procedures

The procedures were performed in a hybrid operating room under general anaesthesia. Transarterial access was established percutaneously or after surgical cut-down. A self-expanding valve prosthesis (Core-Valve, Medtronic Inc., Minneapolis, MN) or a balloon-expandable prosthesis (Edwards SAPIEN, Edwards Lifesciences, Irvine, CA) was used. Rapid right ventricular pacing was performed during balloon dilation for a native aortic valve and at the time of implantation for a balloon-expandable bioprosthetic valve. The position of the prosthetic valve was decided according to the intraoperative multislice computed tomographic findings. The contrast agent used was Iopamidol, which is iodinated and non-ionic and has low osmolarity. All patients were extubated within 6 h of the procedure.

### Definitions and collection of data

The data collected included age, sex, height, BW, the presence of hypertension, hyperlipidaemia, diabetes mellitus, peripheral artery disease, cerebrovascular disease, prior percutaneous coronary intervention, prior coronary artery bypass grafting, chronic obstructive pulmonary disease, SCr, estimated glomerular filtration rate (eGFR), or chronic kidney disease (CKD), STS (Society of Thoracic Surgeons Predictive Risk of Mortality) score, and Logistic EuroSCORE. The SCr level was measured on the day before TAVI and on days 0, 1, 2, 3, 5, and 7 thereafter. The CMV × SCr/BW values were calculated based on the preoperative SCr and BW and the intraoperative CMV.

AKI was defined according to the VARC-2 definition as an absolute reduction in kidney function (for < 7 days) as follows: stage 1, an increase in SCr to 150–199%, an increase in SCr of ≥ 0.3 mg/dl, or urine output < 0.5 ml/kg/h for > 6 h but < 12 h; stage 2, an increase in SCr to 200–299% or urine output < 0.5 ml/kg/h for > 12 h but < 24 h; stage 3, an increase in SCr to ≥ 300% or SCr ≥ 4.0 mg/dl with an acute increase of ≥ 0.5 mg/dl or urine output < 0.3 ml/kg/h for ≥ 24 h or anuria for ≥ 12 h. Patients receiving renal replacement therapy are considered to meet the stage 3 criteria. We did not diagnose AKI based on urine volume. The patient demographics, clinical characteristics, and intraoperative findings were compared between the AKI group and the non-AKI group.

### Statistical analysis

We grouped the patients according to whether they developed postoperative AKI and searched for contributing factors. Categorical variables are presented as the frequency and percentage and were compared using the chi-square or Fisher’s exact test. The normality of distributions was assessed using the Shapiro–Wilk test; normal and skewed continuous variables are presented as the mean ± standard deviation and median (interquartile range), respectively. Continuous variables were compared using the Student’s *t*-test or Mann–Whitney *U* test.

Univariate logistic regression analysis was used to test variables for statistically significant differences. Receiver-operating characteristic (ROC) curve analyses were used to examine the ability of the variables to predict AKI. The prediction performance of each variable was examined by comparing the area under the ROC curves. All tests were two-sided. A p-value < 0.05 was considered statistically significant. All analyses were performed using JMP Pro 13 (SAS Institute Inc., Cary, NC).

### Results

One hundred patients underwent TAVI during the study period. The TA or transsubclavian approach was used in 16 patients and the TF approach in 84. Three patients who underwent TF-TAVI developed intraoperative complications and were excluded, leaving data for 81 patients available for analysis (see Additional file 1: Figure S1). AKI occurred in 7 (8.6%) of these patients and was categorised as grade 1 in 6 (85.7%) and grade 3 in one (14.3%). No patient required renal replacement therapy. The baseline characteristics of the non-AKI (n = 74) and AKI (n = 7) groups are shown in Table 1. The mean age was 84.6 ± 5.1 (range, 64–94) years and 72.8% were women. The mean STS score was 6.9% ± 3.7% and the mean Logistic EuroSCORE was 18.7% ± 10.9%. There was no significant between-group difference in the frequency of CKD. The preoperative SCr value was significantly higher and the preoperative eGFR was significantly lower in the AKI group than in the non-AKI group (1.32 mg/dl vs 0.87 mg/dl, p = 0.0232, and 29.5 ml/min/1.73 m^2^ vs 49.6 ml/min/1.73 m^2^, p = 0.0395, respectively).

**Table 1:** Baseline characteristics of the study population

	Non-AKI (n = 74)	AKI (n = 7)	p-value
Age, years	85.5 (82–88)	83 (81–86)	0.337
Male sex	19 (25.7%)	3 (42.9%)	0.382
Height, cm	147.8 ± 9.8	150.0 ± 7.0	0.564
Body weight, kg	48.2 ± 10.2	44.3 ± 9.3	0.323
Hypertension	59 (79.7%)	6 (85.7%)	1.000
Hyperlipidaemia	41 (55.4%)	5 (71.4%)	0.693
Diabetes mellitus	13 (17.6%)	2 (28.6%)	0.608
PAD	16 (21.6%)	1 (14.3%)	1.000
CVD	18 (24.3%)	1 (14.3%)	1.000
COPD	23 (31.1%)	3 (42.9%)	0.675
CABG	7 (9.5%)	1 (14.3%)	0.531
PCI	12 (16.2%)	3 (42.9%)	0.114
CKD	57 (77.0%)	6 (85.7%)	1.000
SCr, mg/dl	0.87 (0.72–1.15)	1.32 (0.84–1.67)	0.023
eGFR, mL/min/1.73 m^2^	49.6 (37.1–60.0)	29.5 (23.2–54.3)	0.039
EF < 40%	7 (9.5%)	1 (14.3%)	0.531
Euro SCORE, %	16.2 (12.1–22.8)	17.4 (8.4–19.2)	0.556
STS score, %	5.7 (4.3–7.6)	8.4 (6.6–10.6)	0.078

The data are shown as the mean ± standard deviation, number (percentage), or median (interquartile range) as appropriate

AKI: Acute kidney injury; CABG: coronary artery bypass grafting; CKD: chronic kidney disease; COPD: chronic obstructive pulmonary disease; CVD: cerebrovascular disease; EF: ejection fraction; eGFR: estimated glomerular filtration rate; EuroSCORE: European System for Cardiac Operative Risk Evaluation; PAD: peripheral artery disease; STS: Society of Thoracic Surgery

There was no significant between-group difference in operating time, anaesthesia time, intraoperative infusion volume, blood loss, urine volume, transfusion volume, or use of elective percutaneous cardiopulmonary support (Table 2). The CMV was significantly higher in the AKI group than in the non-AKI group (103 ml vs 84 ml, p = 0.025), as was the CMV × SCr/BW value (3.34 vs 1.49, p = 0.0002).

**Table 2:** Perioperative findings in the study population

	Non-AKI (n = 74)	AKI (n = 7)	p-value
Operating time (min)	109 (97–131)	121 (108–229)	0.135
Anaesthesia time (min)	242 (226–273)	293 (240–339)	0.071
Intraoperative fluids (ml)	1832 (1573–2210)	2001 (1612–2312)	1.000
Bleeding (ml)	49 (30–94)	60 (40–100)	0.584
Urine (ml)	700 (395–1243)	500 (330–800)	0.178
Transfusion (ml)	280 (0–560)	280 (280–560)	0.085
CMV (ml)	84 (75–93)	103 (85–121.08)	0.025
CMV × SCr/BW value	1.49 (1.25–2.21)	3.34 (3.00–4.52)	0.0002
Fluoroscopy time (min)	30.5 (24.7–38.6)	30.7 (24.8–89.1)	0.602
PCPS	7 (9.46%)	0 (0.0%)	1.000

The data are shown as the number (percentage) or median (interquartile range) as appropriate

AKI: Acute kidney injury; BW: body weight; CMV: contrast medium volume; PCPS: percutaneous cardiopulmonary support; SCr: serum creatinine

The SCr, eGFR, CMV, and CMV × SCr/BW value were included in the logistic regression analysis. The area under the ROC curve (see Additional file 2: Figure S2) showed that the CMV × SCr/BW value was best able to predict AKI, with an area under the curve of 0.9228 (95% confidence interval 0.650–0.984), a cut-off of 2.99, a sensitivity of 85.7%, and a specificity of 90.4%. The area under the curve for the CMV × SCr/BW value was significantly higher than that for the other variables (Table 3).

**Table 3:** Comparison of areas under the curve

	AUC (95% CI)	p-value
CMV × SCr/BW value	0.9228 (0.6757–0.9856)	Reference
CMV	0.7683 (0.5095–0.9137)	0.0345
SCr	0.7606 (0.5205–0.9029)	0.0230
eGFR	0.7375 (0.4383–0.9100)	0.0193

AUC: Area under the curve; BW: body weight; CI: confidence interval; CMV: contrast medium volume; eGFR: estimated glomerular filtration rate; SCr: serum creatinine

### Discussion

In this study, 7 patients (8.6%) undergoing TF-TAVI for severe aortic stenosis developed AKI; 6 (7.4%) had stage 1 AKI and one (1.2%) had stage 3 AKI according to the VARC-2 criteria. No patient needed renal replacement therapy. Risk factors for AKI included the SCr, eGFR, CMV, and CMV × SCr/BW value. A CMV × SCr/BW value > 2.99 could be considered the threshold value for prediction of AKI during TF-TAVI and for intervention.

In 2012, the endpoint definitions in the VARC-2 consensus document were revised [31] to include a recommendation for the Acute Kidney Injury Network criteria to add urine output in the definition of AKI and the timing for diagnosis of postoperative AKI was extended from 72 h to 7 days. A recent meta-analysis identified New York Heart Association functional class IV, previous CKD, requirement for red blood cell transfusion, previous peripheral artery disease, and a TA approach as strong risk factors for AKI after TAVI according to the VARC-2 definition [33]. Furthermore, the rate of AKI was higher in patients who underwent TA-TAVI than in those who underwent TF-TAVI, as reported previously [21, 34–37]. Another study identified major bleeding to be an important risk factor for AKI and to have a significant impact on outcomes [38].

Our rate of AKI following TAVI is consistent with the observations of Keles et al. [39] and Konigstein et al. [28], who found rates of 7.1% and 16.7% in 70 and 300 patients, respectively. The definition of AKI used (VARC-2 criteria) and the ratio of TF procedures (92.9% and 98%, respectively) in those studies were very similar to those in our study. Consistent with the previous research, we found that the baseline SCr, eGFR, CMV, and CMV × SCr/BW value predicted AKI. Elhmidi et al. [18] and Seiffert et al. [40] identified a correlation between baseline renal function and incidence of AKI after TAVI. Furthermore, Van Linden et al. [27] reported administration of a greater amount of contrast medium to be an independent risk factor for AKI, while Yamamoto et al. [41] identified a relationship between an increment in the dose of contrast medium and an increased prevalence of AKI in their series of 415 consecutive patients who underwent TF-TAVI.

The CMV × SCr/BW value has been established as a criterion for prevention of CIN, and a value > 5.0 was shown to predict post-procedural AKI after percutaneous coronary intervention [11–14]. In our study, a CMV × SCr/BW value > 2.99 was a risk factor for AKI after TAVI. The reason why our threshold CMV × SCr/BW value was smaller than in an earlier coronary angiography study [11] may lie in the difference in the diagnostic criteria used for AKI and CIN. The diagnostic criterion for CIN is an increase in SCr of > 0.5 mg/dl or an increase of > 25% from baseline in the 48–72 h following administration of contrast medium [36]. However, the definition of AKI in the VARC-2 document is based on the Acute Kidney Injury Network classification, i.e., only a slight increase in SCr of 0.3 mg/dl is needed to diagnose the onset of AKI in patients with normal renal function.

In this study, we excluded patients who underwent TA-TAVI and those who had massive bleeding or failure of circulatory dynamics intraoperatively. For the first time, it was possible to identify CMV as a risk factor for AKI after TAVI. Furthermore, although the CMV used was smaller than that in previous studies, the incidence of AKI was comparable. Therefore, renal function and body weight should be taken into account when determining the CMV in, for example, an elderly patient with a small body habitus, which is common in Asian populations. By using the CMV × SCr/BW formula, it is possible to determine the maximum amount of contrast medium that can be used on a case-by-case basis according to preoperative renal function and BW. By determining the maximum dose of the contrast agent, the risk of AKI can be decreased by limiting the CMV, the dilution factor, and the type of contrast agent used. Our findings suggest that avoidance of major complications and reducing the CMV decreases the risk of AKI after TF-TAVI.

## Limitations

This study has several limitations. First, it was small and had a retrospective observational design, so our results may have been affected by unknown cofounders. Second, we did not apply diagnostic criteria based on urine output. Therefore, it is possible that the number of cases of AKI was underestimated. Third, the long-term renal function and outcomes in patients with AKI were not investigated. In a previous study, even a small increase in the baseline creatinine level after TAVI was associated with a worse outcome [25]. The poor prognosis in our patients should encourage better patient selection and management for prevention of AKI.

## Additional files

**Additional file 1: Figure S1.** Flow chart showing patient selection. TA: Transapical; TAVI: transcatheter aortic valve implantation; TF: transfemoral; TS: transsubclavian.

**Additional file 2: Figure S2.** Receiver-operating characteristic curves for predicting acute kidney injury after transfemoral transcatheter aortic valve implantation. AUC: Area under the curve; BW: body weight; CMV: contrast medium volume; Cre: creatinine; eGFR: estimated glomerular filtration rate; SCr: serum creatinine.

## Data Availability

The datasets generated during and/or analysed during the current study are available from the corresponding author on reasonable request.

## Abbreviations

AKI: acute kidney injury
AUC: area under the curve
BW: body weight
CIN: contrast-induced nephropathy
CKD: chronic kidney disease
CMV: contrast medium volume
eGFR: estimated glomerular filtration rate
EuroSCORE: Logistic European System for Cardiac Operative Risk Evaluation
ROC: receiver-operating characteristic
SCr: serum creatinine
STS: Society of Thoracic Surgeons Predictive Risk of Mortality
TA: transapical
TAVI: transcatheter aortic valve implantation
TF: transfemoral
VARC: Valve Academic Research Consortium
