# Supplementary material for: Influence of preoperative serum creatinine level and intraoperative volume of contrast medium on the risk of acute kidney injury after transfemoral transcatheter aortic valve implantation: a retrospective observational study
Source: BMC Res Notes. 2019 Aug 5;12:484. doi: 10.1186/s13104-019-4527-2 (PMC6683543; doi:10.1186/s13104-019-4527-2)
Supplement: Supplementary file 1 — Additional file 1: Figure S1. Flow chart showing patient selection. TA: Transapical; TAVI: transcatheter aortic valve implantation; TF: transfemoral; TS: transsubclavian. [file 13104_2019_4527_MOESM1_ESM.pdf]

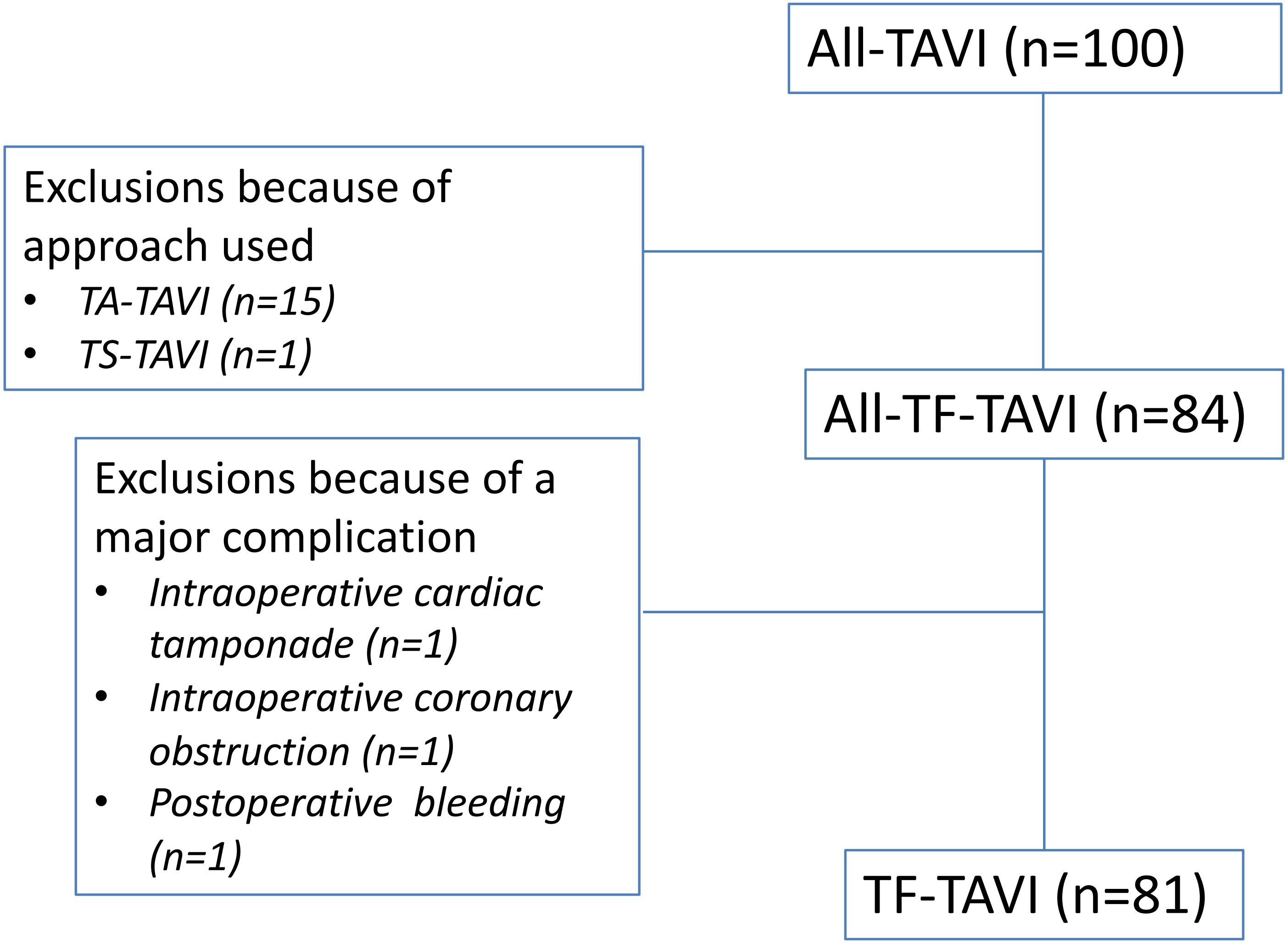
All-TAVI (n=100)
Exclusions because of approach used
TA-TAVI (n=15)
TS-TAVI (n=1)
All-TF-TAVI (n=84)
Exclusions because of a major complication
Intraoperative cardiac tamponade (n=1)
Intraoperative coronary obstruction (n=1)
Postoperative bleeding (n=1)
TF-TAVI (n=81)
