# Supplementary figures and images for: Influence of preoperative serum creatinine level and intraoperative volume of contrast medium on the risk of acute kidney injury after transfemoral transcatheter aortic valve implantation: a retrospective observational study
Source: BMC Res Notes. 2019 Aug 5;12:484. doi: 10.1186/s13104-019-4527-2 (PMC6683543; doi:10.1186/s13104-019-4527-2)

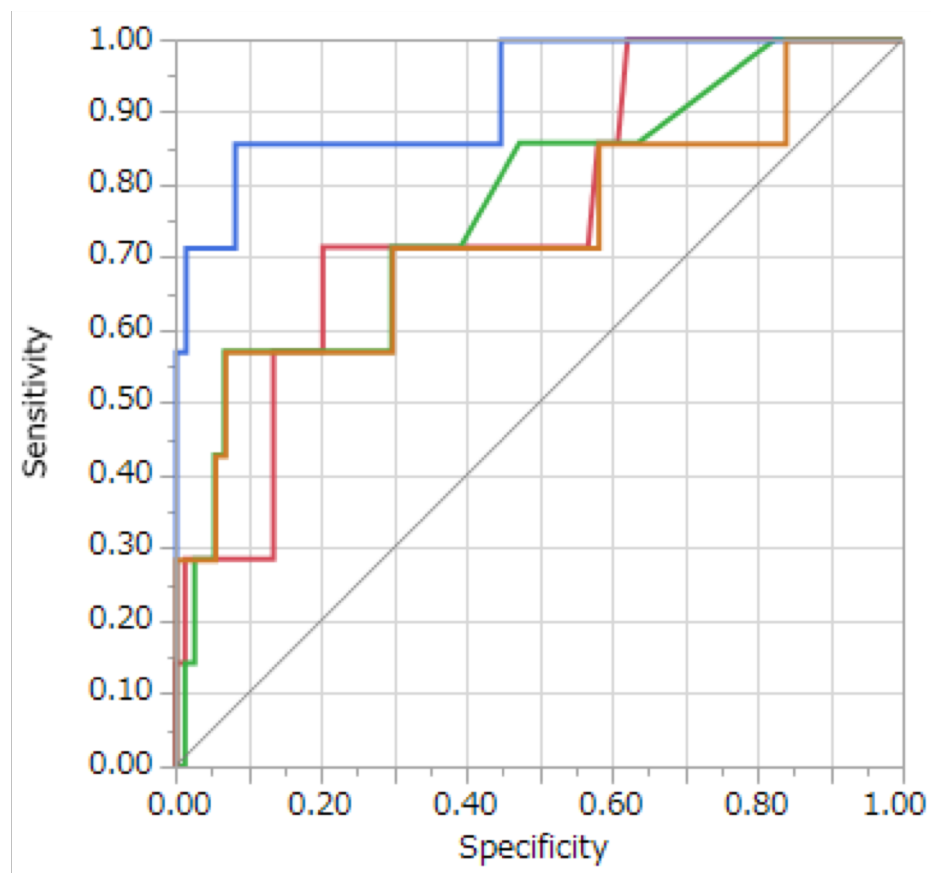

|   |              | AUC    | Cut-off | Sensitivity (%) | Specificity (%) |
|---|--------------|--------|---------|-----------------|-----------------|
| — | SCr          | 0.7606 | 1.19    | 71.4            | 79.3            |
| — | CMV          | 0.7683 | 103     | 57.1            | 92.0            |
| — | eGFR         | 0.7375 | 29.5    | 57.1            | 93.6            |
| — | CMV × SCr/BW | 0.9228 | 2.99    | 85.7            | 90.4            |

Supplement: Supplementary file 2 — Additional file 2: Figure S2. Receiver-operating characteristic curves for predicting acute kidney injury after transfemoral transcatheter aortic valve implantation. AUC: Area under the curve; BW: body weight; CMV: contrast medium volume; Cre: creatinine; eGFR: estimated glomerular filtration rate; SCr: serum creatinine. [file 13104_2019_4527_MOESM2_ESM.pdf]
